# P-701. Etiology, symptoms, and outcomes of viral respiratory tract infections among nursing home residents: Results from multiplex respiratory pathogen testing in the Nursing Home Public Health Response Network, February, 2024 – March, 2025, United States

**DOI:** 10.1093/ofid/ofaf695.913

**Published:** 2026-01-11

**Authors:** Alfonso Hernandez, Tiffany G Harris, Yasin Abul, David Canaday, Christopher J Crnich, Scott Fridkin, Samantha Fuller, Jon P Furuno, Stefan Gravenstein, Steven M Handler, Lindsay B LeClair, Jennifer Meddings, Jennifer Meece, Lona Mody, David A Nace, Paulina Rebolledo, Jennifer L Harcourt, Amanda B Payne, Rachel Slayton, Majerle Reeves, Morgan Katz, Kelly M Hatfield, Alexandra Mellis, Hannah L Kirking, Sujan Reddy

**Affiliations:** CDC, Decatur, Georgia; Abt International, Rockville, Maryland; Brown University, Providence, Rhode Island; VA Northeast Ohio Healthcare System, Cleveland, OH; University of Wisconsin School of Medicine and Public Health, Madison, WI; emory university, Atlanta, Georgia; Abt Global, Rockville, Maryland; Oregon State University, Portland, Oregon; Brown University, Providence, Rhode Island; University of Pittsburgh, Pittsburgh, Pennsylvania; Abt Global, Rockville, Maryland; University of Michigan and the Ann Arbor VA Healthcare System, Ann Arbor, MI; Marshfield Clinic Research Institute, Marshfield, Wisconsin; University of Michigan, Ann Arbor, Michigan; University of Pittsburgh, Pittsburgh, Pennsylvania; Emory University School of Medicine, Emory University Rollins School of Public Health, Atlanta, GA; CDC, Decatur, Georgia; CDC, Decatur, Georgia; Centers for Disease Control and Prevention, Atlanta, GA; CDC, Decatur, Georgia; Johns Hopkins, Stevenson, MD; Centers for Disease Control and Prevention, Atlanta, GA; Centers for Disease Control and Prevention, Atlanta, GA; Coronavirus and Other Respiratory Viruses Division, National Center for Immunization and Respiratory Diseases, CDC, Atlanta, GA; CDC, Decatur, Georgia

## Abstract

**Background:**

The etiology, symptoms, and outcomes of respiratory tract infections (RTI) in nursing home (NH) residents are not well characterized due to limited testing. We describe the epidemiology of RTIs among NH residents in a network of 8 academic sites and 40 affiliated NH.

Figure 1

**Figure 1.**
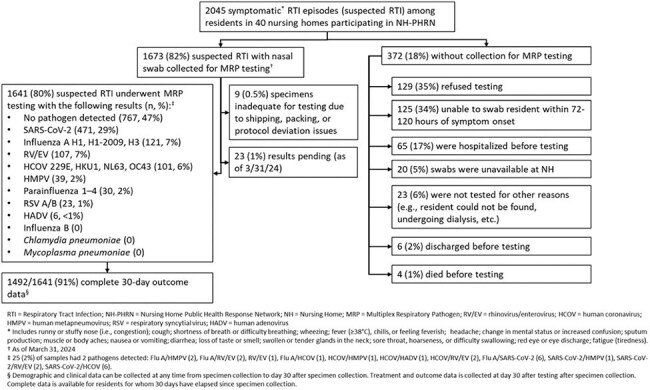
Flow diagram and multiplex respiratory panel testing results of nursing home residents in the Nursing Home-Public Health Response Network, February 2024–March 2025.

Figure 2

**Figure 2.**
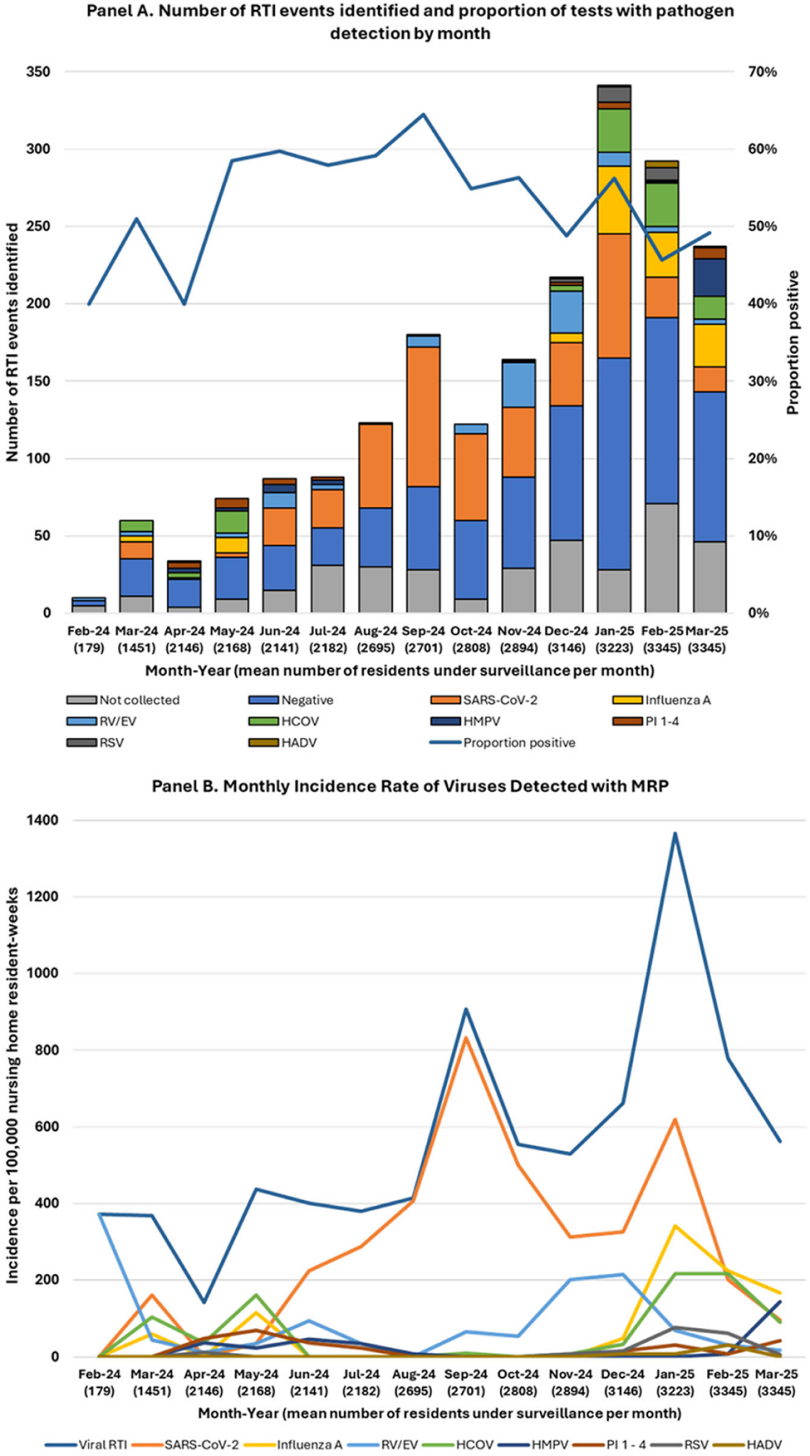
MRP testing results (Panel A) and incidence of viral RTI and select pathogens (Panel B) in 40 nursing homes in the Nursing Home- Public Health Response Network, February 2024 – March 2025, United States. IR = incidence rate HADV= human adenovirus; HCOV = human coronavirus; HMPV = human metapneumovirus; RV/EV = rhinovirus/enterovirus; PI = parainfluenza; RSV = respiratory syncytial virus.

**Methods:**

We conducted RTI surveillance using a multiplex respiratory panel (MRP) test during February 2024 – March 2025. Staff collected anterior nasal swab (NS) specimens from residents with new or worsening respiratory symptoms (suspected RTI) (Figure 1) ≤5 days from symptom onset and collected clinical and outcome data 30 days after testing. NS were tested with the Roche ePlex Respiratory Pathogen Panel 2 (17 viral and bacterial targets) in a central laboratory. We calculated viral rates of RTI per 100,000 resident-weeks. We compared the frequency of symptoms and outcomes by pathogen using Chi-Squared and Fisher’s Exact tests, and 95% confidence intervals (CI) for proportions with Wilson Scores.

**Table 1.**
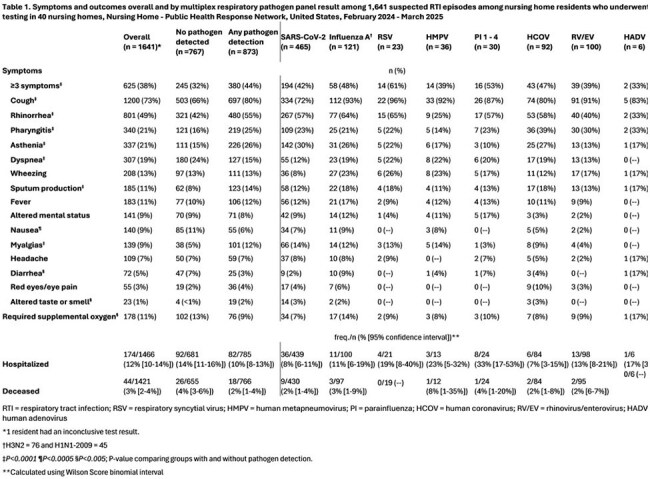
Symptoms and outcomes overall and by multiplex respiratory pathogen panel result among 1,641 suspected RTI episodes among nursing home residents who underwent testing in 40 nursing homes, Nursing Home - Public Health Response Network, United States, February 2024 - March 2025

**Results:**

We identified 2,045 suspected RTIs in 150,151 residents. Testing was done for 1,641 suspected RTIs (80%)(Figure 1). MRP detected ≥1 virus in 874 specimens (53%, range 40-60% per month) (Figure 2, Panel A). Viral RTI rates per 100,000 resident-weeks ranged from 143 in April 2024 to 1365 in January 2025 (Figure 2, Panel B). Rates were highest for SARS-CoV-2 and influenza A (peak 833 and 341 per 100,000 resident-weeks, respectively). Although residents with pathogen detection were more likely to have ≥3 symptoms, cough, rhinorrhea, pharyngitis, sputum production, asthenia, and myalgias than residents without pathogen detection(*P*< 0.0001), symptom frequencies were similar between detected pathogens (Table 1). Among residents with pathogen detection, the proportion with subsequent hospitalization was highest among residents with parainfluenza (33%, 95%CI 17-53%) and human metapneumovirus (HMPV) (23%, 95%CI: 5–32%)(Table 1).

**Conclusion:**

Symptoms could not easily differentiate RTI etiology in the NH population. SARS-CoV-2 was the most frequently detected pathogen, but residents with HMPV or parainfluenza virus had the highest rates of hospitalization and death. Expanding MRP testing in NH could help better understand respiratory pathogen transmission and improve infection prevention and control practices.

**Disclosures:**

Yasin Abul, MD, CDC/ABT: Grant/Research Support|CLARIO: Advisor/Consultant|GSK: Grant/Research Support|Moderna: Grant/Research Support|Seqirus: Grant/Research Support David Canaday, MD, Moderna: Grant/Research Support|Pfizer: Grant/Research Support|Seqirus: Advisor/Consultant|Seqirus: Grant/Research Support|Seqirus: Honoraria Christopher J. Crnich, MD, PhD, Merck: Grant/Research Support Samantha Fuller, MPH, AstraZeneca: Grant/Research Support|CSL Vifor: Grant/Research Support|GlaxoSmithKline: Grant/Research Support Jon P. Furuno, PhD, Merck: Grant/Research Support Stefan Gravenstein, MD, MPH, GSK: Advisor/Consultant|GSK: Grant/Research Support|GSK: Honoraria|Moderna: Grant/Research Support|Novavax: Advisor/Consultant|Novavax: Honoraria|Pfizer: Advisor/Consultant|Pfizer: Grant/Research Support|Pfizer: Honoraria|Sanofi: Advisor/Consultant|Sanofi: Grant/Research Support|Sanofi: Honoraria|Seqirus: Grant/Research Support Lona Mody, MD, MS, Nanovibronix: Grant/Research Support|NIH, CDC, VA: Grant/Research Support|UpToDate: Honoraria Morgan Katz, MD, MHS, Skinclique: Advisor/Consultant

